# Advice on the Management of the Teeth

**Published:** 1850-01

**Authors:** T. B. Hamlin

**Affiliations:** Nashville, Tenn.


### ADVICE ON THE MANAGEMENT OF THE TEETH.

BY T. B. HAMLIN, D. D. S.—*Of Nashville, Tenn.*

The above is the title of a small pamplet published, and we presume distributed among his patients, by Dr. Hamlin. To such it is valuable, containing much which, it properly regarded, would aid in the preservation of the teeth. The better the public are informed on these things, the more will they appreciate good operations, and hence less apt to encourage quackery.

We select the following on Dental Hygiene, as a specimen of the style and character of this pamphlet.—[J. T. Cin. Ed.

Hygiene is the art of preserving health, and implies a knowledge of the influence which certain physical and moral agents are capable of exerting upon the body. The rules of Hygiene must, to a certain extent, have reference to the health of the whole organism, though they are sometimes restricted to a single class of organs. But the sanatory effects resulting from the observance of the rules of health for a particular class of organs, must, of necessity, be more or less limited, unless the rules of the body are, at the same time, perseveringly practiced. But as it constitutes no part of the design of the author of this essay to treat upon general Hygiene, he must confine his remarks to the means necessary for the preservation of the teeth.

Lefoulon, a French writer of some celebrity, in alluding to Dental Hygiene, says, "This branch of our art is more extensive than might be supposed, and its importance requires that we" (referring to the members of the dental profession) "should direct to it our closest attention. How many mutilated persons," says he, "there are, who are afraid to smile, lest they should display the disagreeable void by which their mouths are deformed, and who might have escaped the ugliness which troubles them in all the circumstances of life, if they had taken but a little care—so little, that the recollection of the trifling amount of carefulness required, adds bitterness to their loss."

Few persons, however, are properly instructed in the means necessary for the prevention of disease in these organs, and even Dentists have bestowed too little attention upon this branch of their art. To have badly arranged, diseased, and partially destroyed teeth, is to be constantly exposed to pain, an offensive breath, "repulsive appearance, inability to masticate the most alimentary substances properly," bad general health, and to be deprived to some extent, the pleasure of social enjoyment. To a female, possessed of refined sensibility, whose happiness in some degree at least, depends on her personal charms, it is peculiarly mortifying to have decayed and badly arranged teeth.

It is true, after disease has invaded and made more or less progress in the destruction of the teeth, the services of the Dentist are often sought, but how much better is it to prevent its attacks. The old adage—"an ounce of prevention is better than a pound of cure," is peculiarly applicable to these organs.

There is no part of the body to which preventives can be more efficiently and successfully applied, than to the dental organism. Disease in these parts can, in almost every instance, if taken in time, be completely and permanently arrested. But unfortunately, the aid of the Dentist, in too many instances, is not sought, until it has committed such fearful ravages, that his skill can only palliate, without effecting a permanent cure. But in view of the importance of the preservation of the health of the teeth and gums, we would urge upon the heads of the families in which we exercise the duties of our profession, the necessity of a constant observance of such hygenic rules as may be required for the accomplishment of this object, and that this can be done, a long practice not limited by localities, has fully satisfied the writer; and it would afford him more pleasure to be instrumental in securing the observance of these rules, than any pecuniary gain which he could possibly derive from their neglect.

The hygenic treatment of the teeth recommended by Dr. L. S. Parmly, is, in the opinion of the writer, the best that has ever been suggested. It is predicated upon the fact that caries of the teeth is the result of the action of external corrosive agents, such as putrid foreign matter and vitiated saliva, and consists in cleansing the teeth thoroughly and regularly four or five times a day, with waxed floss silk. By this means the approximal surfaces of the teeth are reached, and all foreign and vitiated matter, which is constantly accumulating here, removed.

But in addition to the use of floss silk, a suitable brush should be used several times a day, say in the morning immediately after rising, and after each meal, and before retiring at night. The frequent use of the brush is necessary, not only for the removal of the remains of alimentary substances lodged in the indentations of the teeth, and along the margins of the gums, but also for the removal of the clammy mucus which accumulates upon and adheres to these organs, and to prevent the formation of salivary calculus, commonly called tartar.

When, from an acidulated condition of the fluids of the mouth, or other cause, the teeth become discolored or stained, their natural whiteness and brilliancy should be restored by the use of a suitable powder, one which will effect the object by its *mechanical* action, or an argelacious tooth polisher, as recommended by Dr. L. S. Parmly. All acids are hurtful to the teeth, as has been conclusively demonstrated by Prof. Westcott by a series of experiments.

But to derive the full benefit of the use of the foreign means, their use should be commenced in early life, as soon as the temporary or milk teeth emerge from the gums, and regularly and perseveringly persisted in. Cleanliness of the teeth, especially such as are of a soft texture, is indispensable to their preservation, and the health of the gums.

Some may regard the use of the means here recommended as troublesome, and to avoid which, permit particles of food and other extraneous matter to remain between their teeth and in their interstices, until they putrify or are decomposed, contaminating the atmosphere they breathe, and rendering it offensive to all who approach them, while they are scrupulously particular with regard to the cleanliness of their face and hands.

If sloth or negligence the task forbear
Of making cleanliness a daily care;
If fresh ablution, with the morning sun,
Be quite forborne, or negligently done;
In dark disguise, insidious tartar comes,
Incrusts the teeth and irritates the gums,
Till vile deformity usurps the seat,
Where smiles should play and winning graces meet,
And foul disease pollutes the fair domain,
Where health and purity should ever reign.—*Dentologia.*

Finally, the importance of keeping the teeth constantly clean, as a preventive of caries and a diseased condition of the gums, is now universally conceded and strenuously advocated by all well-informed practitioners of Dental Surgery.

